# Sex-specific effects of maternal dietary carbohydrate quality on fetal development and offspring metabolic phenotype in mice

**DOI:** 10.3389/fnut.2022.917880

**Published:** 2022-07-22

**Authors:** G. Jean Campbell, Sophie G. Lucic Fisher, Amanda E. Brandon, Alistair M. Senior, Kim S. Bell-Anderson

**Affiliations:** ^1^Charles Perkins Centre and School of Life and Environmental Sciences, University of Sydney, Sydney, NSW, Australia; ^2^Charles Perkins Centre and Sydney Medical School, University of Sydney, Sydney, NSW, Australia

**Keywords:** maternal diet, glycemic index, carbohydrate quality, metabolism, mice

## Abstract

**Objectives:**

*In utero* glycemia is an important determinant of fetal growth. Women with gestational diabetes are more likely to deliver large-for-gestational age babies that are at increased risk for obesity. The maternal nutritional state modulates the development of offspring biological systems during the critical periods of gestation and lactation. Carbohydrate typically contributes most of the dietary energy, however, there are very few mechanistic studies investigating the effects of maternal dietary carbohydrate quality on fetal and offspring outcomes. Therefore, we sought to investigate the direct effects of maternal carbohydrate quality on sex-specific offspring metabolic programming.

**Methods:**

Female C57BL/6 mice were fed one of five isocaloric diets: four high-sugar diets based on glucose, sucrose, isomaltulose or fructose (all containing 60% energy as carbohydrate), or a standard, minimally processed, chow diet, and were mated with chow-fed males. Half of the dams were sacrificed for fetus dissection and placental collection, with the remaining giving live birth. All dams were metabolically profiled before and during pregnancy, and pups were similarly profiled at 12 weeks of age.

**Results:**

Overall, glucose-fed dams were heavier and fatter than chow or isomaltulose-fed dams. Female fetuses from glucose and isomaltulose-fed mothers weighed less and had smaller livers, than those from chow-fed mothers, with isomaltulose-fed female fetuses also having decreased placental mass. In contrast, male fetuses responded differently to the maternal diets, with heart mass being significantly increased when their mothers were fed fructose-containing diets, that is, sucrose, isomaltulose and fructose. High-sugar fed female offspring weighed the same, but were significantly fatter, than chow-fed offspring at 12 weeks of age, while glucose and isomaltulose-fed male pups displayed a similar phenotype to their mothers’.

**Conclusion:**

While both glucose and isomaltulose diets constrained fetal growth in females, only placentas from isomaltulose-fed dams were significantly smaller than those from chow-fed mothers, suggesting the mechanisms through which fetal growth is reduced may be different. Female fetuses of isomaltulose-fed mothers were also lighter than sucrose-fed fetuses suggesting the glycemic index, or rate of glucose digestion and absorption, may be an important factor in determining nutrient availability to the growing fetus.

## Introduction

Diet is the major environmental factor impacting health and contributing to non-communicable disease. Over the past three decades there has been a substantial shift globally toward greater consumption of refined, processed foods at the expense of fresh, whole foods similar to those that humans evolved to consume (1–3). Advances in food processing and public health recommendations to eat low-fat foods has seen an increase in shelf-stable, refined carbohydrate-rich foods and a significant change in the nature of dietary carbohydrates in the global food supply (4). Carbohydrate quality has been implicated in links with health and disease (5–10).

Dietary guidelines recommend half of dietary energy comes from carbohydrate (11, 12). Carbohydrate quality of food is largely determined by type, processing, and source. The consumption of whole grains and fiber, generally considered high quality carbohydrates, has decreased over time while intake of rapidly digestible carbohydrate has increased (13). Consequently, today more than 70% of dietary carbohydrate consumed is absorbed as the glucose moiety from digestion of starch and refined sucrose (and similar products), the rest a mix of mainly fructose and galactose (14).

One of the most popular methods of quantifying carbohydrate quality is the glycemic index (GI). GI is a physiological method of assessing the impact of a carbohydrate containing food on blood glucose during the postprandial state (15). Foods with a low GI maintain a more stable blood glucose over this time period, whereas high GI foods produce a rapid spike in blood glucose. Low GI diets are associated with improved health outcomes, including weight management, better insulin sensitivity, and lowered risk of type 2 diabetes mellitus (16). However, fructose, despite its low GI, has been associated with obesity and poor cardiometabolic health (17, 18). As GI is a very simple measure, it can be easily clouded by other factors such as fiber and macronutrient content, energy density, and level of processing.

In this study we aimed to test and compare how sugars of different GIs affect metabolic health outcomes in female mice and their offspring. The diets were designed to determine direct effects of GI (sucrose vs. isomaltulose), as well as to tease out the effects of different saccharides that contribute to simple carbohydrate intake (glucose vs. fructose), independently of energy density, macronutrient composition and wholegrain and fiber content, as other markers of carbohydrate quality. In previous studies, we measured the GI of standard laboratory chow and these high-sugar diets *in vivo* in mice, emulating the GI testing procedure utilized in humans. We showed that non-meat, minimally processed, rodent chow is relatively high GI. The GIs of the different sugar diets occur in the same rank order as in humans with glucose being the highest, followed by sucrose, isomaltulose, and fructose as the lowest (19).

Sucrose and isomaltulose are both disaccharides of glucose and fructose. However, due to different bonds between the saccharides, sucrose is cleaved faster and absorbed quicker by the enterocyte, leading to a more rapid rise in blood glucose after digestion than when compared to isomaltulose (20). This comparison of sucrose and isomaltulose response is particularly interesting as high consumption of fructose containing sugars is repeatedly associated with increased obesity and fatty liver disease. However, it appears that slowing down the rate of fructose absorption, specifically when ingested in the form of isomaltulose, may help limit the complications associated with fructose intake (21, 22). Isomaltulose is a natural sugar but most isomaltulose in commercial food is made by an industrial conversion of sucrose and marketed as Palatinose™. Isomaltulose has the potential to improve the food supply as a healthier alternative sweetener to sucrose (22).

While a large body of evidence indicates the effects of high-sugar consumption in obesity, few have explored the generational consequences. The theory of Developmental Origins of Health and Disease describes a concept where maternal nutritional and metabolic status can influence risk of non-communicable disease in offspring (23). Early studies in this field have observed the consequences of maternal undernutrition. However, dietary patterns correlated with overnutrition and obesity have been less well studied. Typically, obesogenic diets used in rodent studies of overnutrition are comprised of a high-fat content, and are often high in sucrose as well. Interventional mouse studies as well as observational studies in human populations have shown excessive body weight at the start of pregnancy is associated with gestational diabetes, preeclampsia, and fetal adiposity (24). Offspring from over nourished mothers are also predisposed to similar metabolic issues as their mothers later in life, such as adult obesity, type 2 diabetes, and cardiovascular disease (25). The effects of maternal sugar consumption on health and disease as it relates to carbohydrate quality and GI requires greater investigation. Although sucrose consumption has been slowly declining over the past 10 years, refined high GI starches and sugars, digested as glucose, have increased (14). *In utero* exposure over several generations may therefore create a vicious cycle of increasing obesity at childbearing age (26). It is therefore important to understand the generational consequences of rapid vs. slowly absorbed carbohydrate consumption.

The aim of the current study was to examine the metabolic effects of a maternal diet varying in saccharide composition and rate of glucose absorption, or GI, on fetal and offspring metabolic outcomes with particular focus on body composition, relative to chow-fed reference mice. To do this, we conducted a study that was segmented into two parts to address two key questions.

Part 1 was designed to investigate the impact of the GI alone of refined carbohydrate diets on metabolic health and development of offspring, using diets comprehensively controlled for all other factors. This involved the direct comparison of a sucrose-based and isomaltulose-based diet, which are identical in every way, excepting the rate of digestion of the respective sugars. We hypothesized that the isomaltulose-based diet would lead to improved metabolic parameters in the offspring.

In Part 2 we directly compared a glucose- and fructose-based diet. We hypothesized that fructose, despite being much lower GI, is not a healthy alternative to glucose and that both diets would be detrimental to metabolic health and offspring development.

## Materials and methods

### Mice and diets

Forty-eight 6-week-old female and 10 12-week-old male C57BL6/J mice were purchased from Australian BioResources (NSW, Australia). All mice were housed in the animal house at the Charles Perkins Centre at the University of Sydney in a 12 h light-dark cycle at 22°C and 60% humidity. After 1-week acclimatization on standard non-meat chow *ad libitum*, mice were randomized to one of five diets also *ad libitum* (Table 1). The same chow-diet mice were used as the reference group for both studies, which were performed at the same time, and were therefore included in both analyses. Chow was purchased from Specialty Feeds (WA, Australia; Supplementary material) while the remaining diets were made and pelleted in-house using a pellet-press (Parr 2811 Pellet Press, Parr Instrument Company, IL, United States) to create pellets approximately 1 cm in diameter and 1 g in weight. The energy density of the diets was determined by bomb calorimeter (Parr Instrument Company, IL, United States). All diets were approximately 64% carbohydrate, 22% protein and 14% fat as a proportion of the total dietary energy (Table 1). After a minimum of 6 weeks on the diets, mice were mated with age-matched chow-fed males. Half the dams were euthanized at day E18 of pregnancy, as inferred from timed-mating, for collection of fetuses and placentas, while the remaining dams went to term and were euthanized at 30 weeks of age. Pups were weaned at 3 weeks of age and remained on the maternal diet *ad libitum* until euthanized at 12 weeks of age. All mice were euthanized following a 4–5 h fast with plasma and tissues collected, snap frozen in liquid N_2_ and stored at –80°C. Fetal organs were dissected in phosphate buffered saline, weighed, and snap frozen in liquid N_2_.

**TABLE 1:** Diet composition.

g/kg	Chow	Glucose	Fructose	Sucrose	Iso- maltulose
Maltodextrin		120	120	120	120
Glucose		480	–	–	–
Sucrose		–	–	480	–
Isomaltulose		–	–	–	480
Fructose		–	480	–	–
Calcium caseinate		200	200	200	200
Safflower oil		70	70	70	70
Bran		50	50	50	50
Mineral mix		45	45	45	45
Gelatin		15	15	15	15
Vitamin mix		13	13	13	13
Choline bitartrate		4	4	4	4
DL-Methionine		3	3	3	3
Carbohydrate %	65	64.2	64.2	64.2	63.3
Protein %	23	21.7	21.7	21.7	22.2
Fat %	12	14.1	14.1	14.1	14.5
Total Energy (kJ/g)	17.0 ± 0.05	16.6 ± 0.2	17.0 ± 0.1	17.1 ± 0.05	16.7 ± 0.1

Ingredient composition for in-house diets based on the standard AIN-93G diet, and macronutrient contribution to total energy for all mouse diets. Total Energy is displayed as mean ± SEM. Further information on dietary ingredients is included in Supplementary Materials.

All animal procedures were approved by the University of Sydney Animal Ethics Committee and complied with the Australian Code for the Care and Use of Animals for Scientific Purposes (27).

### Metabolic phenotyping

Dams were metabolically phenotyped before, and at day E11 of pregnancy, with pups being similarly screened at 12 weeks. After a 4–5 h fast, mice underwent an oral glucose tolerance test (OGTT) with 200 μL of 25% (w/v) glucose administered *via* oral gavage. Blood was collected from the tail with glucose levels measured using an Accu-Chek glucometer (Roche Diabetes Care, Switzerland) before gavage and at 15, 30, 45, 60, 90, and 120 min following. Tail blood insulin was also measured at basal, 15, 30, and 120 min using an Ultrasensitive Insulin ELISA Kit (Crystal Chem, IL, United States). Two to 3 days following an OGTT, mice were placed in an EchoMRI 900 machine (EchoMRI, TX, United States) to obtain measurements of body lean and fat mass.

Energy expenditure and respiratory quotient (RQ) were determined from oxygen consumption rate (VO_2_) and carbon dioxide production rate (VCO_2_) measured under consistent temperature (22°C) using an indirect calorimetry system (Promethion, Sable System International, NV, United States). VO_2_ and VCO_2_ of individual mice in home-style cages was measured at 5 min intervals over a 2–3 day period. Mice had *ad libitum* access to food and water and wheel. The RQ was calculated by dividing VCO_2_ by VO_2_. Physical activity was calculated from voluntary locomotion in individual mice in the metabolic cages measured by X, Y, and Z beam breaks and wheel revolutions.

### Biochemical analysis

Insulin response to an OGTT and basal insulin levels at sacrifice were determined using an Ultrasensitive Insulin ELISA Kit (Crystal Chem, IL, United States) with 10 μL of tail blood or 5 μL plasma, respectively, following the manufacturer’s instructions. Pup plasma leptin at sacrifice was also measured using a Mouse Leptin ELISA Kit (Crystal Chem, IL, United States) following manufacturer’s instructions.

Liver triglycerides were extracted using the method of Folch et al. (28). In brief, 20–30 mg of tissue were ruptured in 4 mL 2:1 chloroform: methanol in a Minibeadbeater-24 (Daintree Scientific, TAS, Australia) and the solution left to extract on a roller for 2 nights. 0.5 volumes of 0.6% (w/v) NaCl was added to aid phase separation and the organic phase removed *via* glass pipette into a glass vial. Lipids were then dried under nitrogen gas at 37°C and resuspended in absolute ethanol. Samples were then loaded onto a microplate, dried at 37°C for 20 min, and quantified *via* an enzymatic colorimetric assay (Triglyceride GPO-PAP reagent, Roche Diagnostics Australia, NSW, Australia). Plasma triglycerides were measured directly from blood plasma by an enzymatic colorimetric assay as above.

### Determination of fetal genetic sex

Fetuses were sexed by genotyping for the *Sry* gene on the Y chromosome using placental DNA extracted using the DNeasy Blood and Tissue Kit (Qiagen, Hilden, Germany) and amplified with the *Taq* DNA Polymerase Core Kit (Qiagen, Hilden, Germany). The primers used were F: TTGTCTAGAGAGCATGGAGGGCCA, R: CACTC CTCTGTGACACTTTAGCC.

### Statistics

Data was analyzed and graphed in R (R Core Team, Vienna, Austria). All comparisons for dam data were determined using ANOVAs in combination with pairwise *t*-tests. To test for the correlation of conception weight vs. total pregnancy weight gain, a linear model was used where significance was determined using the F-statistic. Two-way ANOVAs were used to test for changes in body weight and glucose or insulin area under the curve (AUC) over time. All differences between offspring were determined by a linear mixed effects model adjusted for litter as a random effect, using the R package lme4 and function lmer(). Two-way ANOVAs were also used to test for changes in pup body weight over time. Data are displayed as mean ± SEM with outliers being removed if they were more than 2 standard deviations away from the arithmetic mean.

## Results

### Dams

#### Part 1 (sucrose vs. isomaltulose)

##### Body weight

**FIGURE 1:**
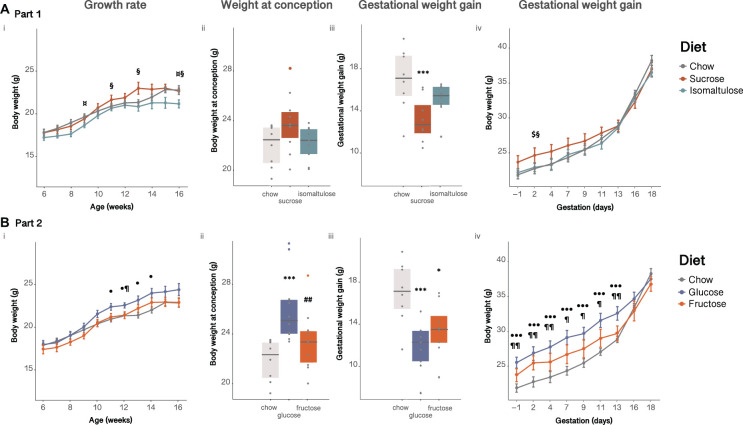
Dam body weight **(A)** in Part 1 and **(B)** in Part 2: (i) pre-pregnancy, (ii) at conception, (iii) gained during pregnancy, (iv) during pregnancy. *n* = 8–10 per diet. Line graphs depict data as mean ± SEM. *p < 0.05 vs. chow; ***p < 0.001 vs. chow; ^##^*p* < 0.01 vs. glucose; ^$^*p* < 0.05 sucrose vs. chow; ¤*p* < 0.05 isomaltulose vs. chow; § *p* < 0.05 sucrose vs. isomaltulose; ^⋅^*p* < 0.05 glucose vs. chow; ^⋅⋅⋅^*p* < 0.001 glucose vs. chow; ¶ *p* < 0.05 glucose vs. fructose; ¶¶ *p* < 0.01 glucose vs. fructose.

All dams steadily increased body weight for the first 6 weeks after being placed on the diets, with sucrose-fed dams becoming significantly heavier than isomaltulose-fed dams from 11 weeks of age, equivalent to 4 weeks on their respective experimental diets (*p* < 0.05; Figure 1Ai). A two-way ANOVA showed a significant effect of diet, age, and the interaction of diet and age, on body weight prior to pregnancy (diet, age *p* < 0.001; interaction *p* < 0.01). There were no significant differences in conception weight between diets (sucrose vs. chow *p* = 0.05; Figure 1Aii). All dams increased body weight throughout pregnancy (Figures 1Aiii,iv). However, sucrose-fed dams gained significantly less weight than chow-fed dams (*p* < 0.001). Adjusting pregnancy weight gain data for litter size and weight using a mixed linear model revealed that both sugar-diet mice groups gained significantly less pregnancy weight than chow-fed mice, (adjusted data not shown; *p* < 0.01 for both). During pregnancy, there was a significant effect of diet, time point, and the interaction of diet and time point, on body weight (diet *p* < 0.05; time point *p* < 0.001; interaction *p* < 0.01).

##### Body fat and triglyceride analyses

While there were no significant differences in percentage body fat between diet groups at preconception, during pregnancy sucrose-fed dams had significantly increased adiposity compared to chow-fed dams (*p* < 0.05; Table 2). Isomaltulose-fed dams were the only group to significantly increase body adiposity from preconception to mid-pregnancy (*p* < 0.05). Mirroring adiposity, the only significant difference in percentage lean mass between diet groups was a comparative decrease in sucrose-fed dams compared to chow-fed dams during pregnancy, with no differences prior to pregnancy (data not shown; *p* < 0.01). However, all three diet groups showed a decrease in percentage lean mass from preconception to pregnancy (chow, isomaltulose *p* < 0.001; sucrose *p* < 0.05).

**TABLE 2:** Dam adiposity, triglyceride levels, and glucose homeostasis before and during pregnancy in Part 1 (sucrose vs. isomaltulose).

	*Timepoint*	Chow	Sucrose	Isomaltulose
Total body fat (%)	*Preconception*	11.2 ± 0.9	14.2 ± 1.7	11.2 ± 1.0
Total body fat (%)	*Mid-gestation*	12.7 ± 0.7	17.3 ± 1.5*	15.2 ± 1.2^†^
Subcutaneous WAT mass (g)	*30 weeks of age*	0.28 ± 0.02	0.64 ± 0.10**	0.40 ± 0.04^
Ovarian WAT mass (g)	*30 weeks of age*	0.31 ± 0.03	0.71 ± 0.21	0.46 ± 0.10
Retroperitoneal WAT mass (mg)	*30 weeks of age*	54 ± 9	145 ± 44	107 ± 26
Intrascapular BAT mass (mg)	*30 weeks of age*	57 ± 5	106 ± 14*	79 ± 14
Plasma triglyceride (μmol/μl)	*At term*	2.5 ± 0.2	1.6 ± 0.2**	1.3 ± 0.2**
Plasma triglyceride (μmol/μl)	*30 weeks of age*	2.3 ± 0.1	1.1 ± 0.3**	1.8 ± 0.2
Liver triglyceride (μmol/g)	*At term*	27 ± 5	32 ± 3	30 ± 4
Liver triglyceride (μmol/g)	*30 weeks of age*	25 ± 4	55 ± 7^‡⁣‡^	43 ± 1
Basal blood glucose (mM)	*Preconception*	8.9 ± 0.4	9.7 ± 0.5*	8.3 ± 0.3^^
Basal blood glucose (mM)	*Mid-gestation*	9.2 ± 0.3	9.4 ± 0.4	9.8 ± 0.2^†⁣†⁣†^
Fasting plasma insulin (ng/ml)	*Preconception*	0.3 ± 0.05	0.5 ± 0.09	0.4 ± 0.06
Fasting plasma insulin (ng/ml)	*Mid-gestation*	0.7 ± 0.08^†⁣†^	1.0 ± 0.21	1.1 ± 0.14^†⁣†^
30 min insulin (ng/ml)	*Preconception*	0.6 ± 0.08	0.9 ± 0.17	0.6 ± 0.07
30 min insulin (ng/ml)	*Mid-gestation*	1.0 ± 0.12^†^	1.4 ± 0.23	1.1 ± 0.16^†^

Dam percentage fat mass, basal fasting blood glucose and plasma insulin, and 30 min insulin in response to an oral glucose tolerance test before and during pregnancy n = 8–10 per diet; dam adipose depot weights at 30 weeks of age n = 4–6 per diet; dam triglyceride levels in plasma and liver at day E18 of pregnancy or 30 weeks of age n = 3–5 per diet per timepoint. Data presented as mean ± SEM. *p < 0.05 vs. chow; **p < 0.01 vs. chow; ^p < 0.05 vs. sucrose; ^^p < 0.01 vs. sucrose; ^†^p < 0.05 vs. preconception for the same diet; ^†⁣†^p < 0.01 vs. preconception for the same diet; ^†⁣†⁣†^p < 0.001 vs. preconception for the same diet; ^‡⁣‡^p < 0.01 vs. at term for the same diet.

WAT, white adipose tissue; BAT, brown adipose tissue.

Expressed as absolute tissue weight, sucrose-fed dams at 30 weeks of age had larger subcutaneous white adipose tissue (WAT) depots than both chow and isomaltulose-fed mice (chow *p* < 0.01; isomaltulose *p* < 0.05; Table 2). Sucrose-fed dams also had significantly larger brown adipose tissue (BAT) depots than chow-fed dams (*p* < 0.05). There were no significant differences in ovarian or retroperitoneal WAT between diet groups.

Sucrose and isomaltulose-fed mice had lower plasma triglyceride levels than chow-fed mice at day E18 of pregnancy, and sucrose-fed mice had lower plasma triglyceride levels than chow-fed mice at 30 weeks of age (*p* < 0.01 for all three comparisons; Table 2). There were no significant differences in plasma triglyceride levels from day E18 of pregnancy to at 30 weeks of age for any diet. There were also no significant differences between diet groups in liver triglyceride levels at the end of pregnancy or at 30 weeks of age. Only sucrose-fed dams had significantly more liver triglyceride content at 30 weeks of age compared to at term (*p* < 0.01).

##### Energy expenditure, respiratory quotient, and physical activity

**FIGURE 2:**
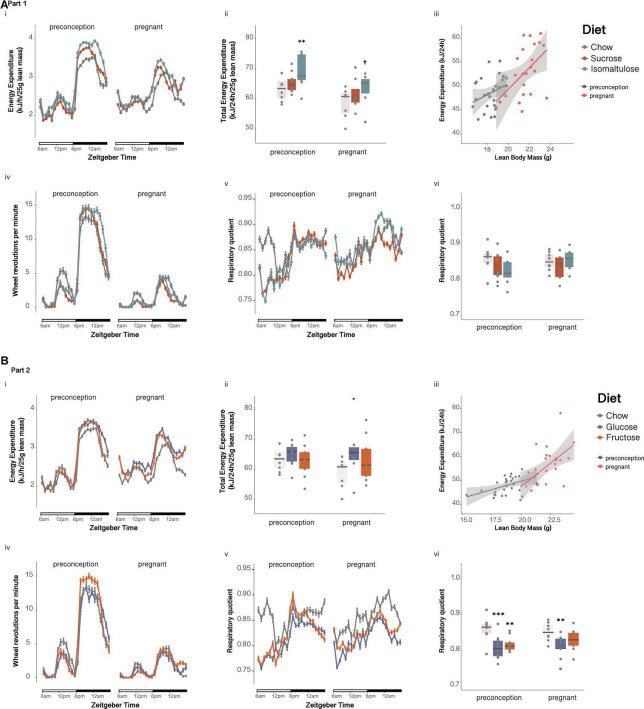
Metabolic phenotyping of dams before and during pregnancy **(A)** in Part 1 and **(B)** in Part 2: Dam (i) energy expenditure over time, (ii) total energy expenditure, (iii) correlation of energy expenditure and lean mass, (iv) wheel revolutions, (v) respiratory quotient over time, and (vi) mean respiratory quotient over 24 h. *n* = 8–10 per diet. Line graphs depict data as mean ± SEM. **p < 0.01 vs. chow; ***p < 0.001 vs. chow; ^†^*p* < 0.05 vs. preconception for the same diet.

Preconception, isomaltulose-fed dams expended more energy per unit of lean mass than chow-fed dams over 24 h (*p* < 0.01; sucrose vs. isomaltulose *p* = 0.052; Figures 2Ai,ii). Mean 24 h energy expenditure was significantly reduced in pregnancy only in isomaltulose-fed dams (*p* < 0.05; chow preconception vs. pregnancy *p* = 0.058). Wheel running was significantly decreased during pregnancy across all three diet groups (*p* < 0.001 for all; Figure 2Aiv), but there were no significant differences in wheel running between diets at either timepoint (preconception: isomaltulose vs. chow *p* = 0.051). There were also no significant differences in mean 24 h RQ between diets at either timepoint, or between timepoints for any diet group (preconception: isomaltulose vs. chow *p* = 0.058; Figures 2Av,vi).

##### Glucose tolerance

**FIGURE 3:**
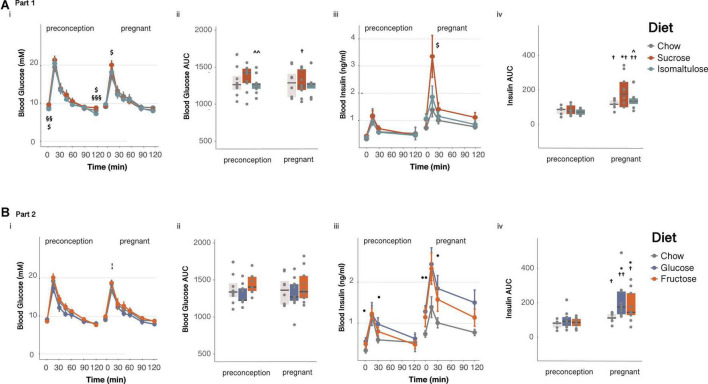
Glucose homeostasis in dams **(A)** in Part 1 and **(B)** in Part 2: Dam response to an oral glucose tolerance test before and during pregnancy (i) glucose over time, (ii) glucose AUC, (iii) insulin over time and, (iv) insulin AUC. *n* = 8–10 per diet. Line graphs depict data as mean ± SEM. *p < 0.05 vs. chow; ^*p* < 0.05 vs. sucrose; ^ ^*p* < 0.01 vs. sucrose; ^$^*p* < 0.05 sucrose vs. chow; §§ *p* < 0.01 sucrose vs. isomaltulose; §§§ *p* < 0.001 sucrose vs. isomaltulose;^⋅^ *p* < 0.05 glucose vs. chow; ^⋅⋅^*p* < 0.01 glucose vs. chow; ¦*p* < 0.05 fructose vs. chow; ^†^*p* < 0.05 vs. preconception for the same diet; ^†⁣†^*p* < 0.01 vs. preconception for the same diet.

Sucrose-fed dams had higher basal blood glucose and higher glucose incremental AUC in response to an OGTT compared to isomaltulose-fed mice, in addition to a higher basal glucose than chow-fed mice, at preconception (basal: chow *p* < 0.05, isomaltulose *p* < 0.01; AUC: chow *p* = 0.058, isomaltulose *p* < 0.01; Figures 3Ai,ii and Table 2). Sucrose-fed dams had higher 120 min blood glucose levels compared to chow and isomaltulose-fed dams at preconception (chow *p* < 0.05; isomaltulose *p* < 0.001). During pregnancy, sucrose-fed dams also had higher 15 min blood glucose levels compared to chow-fed dams (*p* < 0.05). There were no other significant differences between diets during pregnancy. Isomaltulose-fed mice showed increased basal blood glucose from preconception to pregnancy (*p* < 0.001). Sucrose-fed dams had decreased AUC and tended to have reduced 120 min glucose levels from preconception to pregnancy (AUC *p* < 0.05; 120 min *p* = 0.059). Chow-fed dams tended to have reduced 15 min peak glucose levels from preconception to pregnancy (*p* = 0.055). As determined by a two-way ANOVA, there was a significant effect of diet, but not time point, nor the interaction between diet and time point, on dam glucose AUC from preconception to pregnancy (*p* < 0.05).

There were no significant differences in insulin response to an OGTT between diets at preconception. Chow and isomaltulose-fed mice increased basal blood insulin from preconception to pregnancy, and all diet groups increased insulin AUC during an OGTT from preconception to pregnancy (basal: chow, isomaltulose *p* < 0.01, sucrose *p* = 0.053; AUC: chow, sucrose *p* < 0.05, isomaltulose *p* < 0.01; Figures 3Aiii,iv and Table 2). Compared to preconception, pregnancy increased blood insulin levels at 30 and 120 min during an OGTT in isomaltulose-fed mice, increased 30 min insulin in chow-fed mice, and increased 120 min insulin in sucrose-fed mice (15 min: sucrose *p* = 0.051; 30 min: chow, isomaltulose *p* < 0.05; 120 min: sucrose, isomaltulose *p* < 0.05). There were few significant differences between diet groups with sucrose-fed mice having increased 15 min blood insulin levels compared to chow-fed mice, and increased insulin AUC compared to both chow and isomaltulose-fed mice, during pregnancy (*p* < 0.05 for all three comparisons). There was a significant effect of both diet and time point, but not the interaction of diet and time point, on dam insulin AUC from preconception to during pregnancy, as determined by a two-way ANOVA (diet *p* < 0.05; time point *p* < 0.001).

#### Part 2 (glucose vs. fructose)

##### Body weight

As in Part 1, all dams steadily increased body weight over time. Glucose-fed dams were significantly heavier from 11 weeks of age until 14 weeks of age, equivalent to 4–7 weeks on the diet, compared to chow-fed dams (*p* < 0.05; Figure 1Bi). As shown by a two-way ANOVA, there was a significant effect of diet, age, and the interaction of diet and age, on dam body weight prior to pregnancy (*p* < 0.001 for all three comparisons). At conception, glucose-fed mice were heavier than chow and fructose-fed mice (chow *p* < 0.001, fructose *p* < 0.01; Figure 1Bii). Similar to Part 1, all dams increased body weight throughout pregnancy (Figures 1Biii,iv). Despite glucose-fed dams weighing more than both other diet groups for the first two thirds of pregnancy, glucose-fed mice gained less weight overall during pregnancy than chow-fed mice (*p* < 0.001). Fructose-fed dams also gained less pregnancy weight that chow-fed dams (*p* < 0.05). After adjustment for litter size and weight, both glucose and fructose-fed dams still gained significantly less pregnancy weight than chow-fed mice (adjusted data not shown; glucose *p* < 0.001; fructose *p* < 0.05). Additionally, glucose-fed dams also gained less pregnancy weight under the linear mixed model than fructose-fed dams (*p* < 0.05). Similar to Part 1, there was a significant effect of diet, time point, and the interaction of diet and time point, on body weight during pregnancy (diet, time point *p* < 0.001; interaction *p* < 0.05).

##### Body fat and triglyceride analyses

Glucose-fed dams had a higher percentage of body fat than chow-fed dams both before and during pregnancy, and fructose-fed dams during pregnancy (preconception: chow *p* < 0.01; pregnancy: chow *p* < 0.001, fructose *p* < 0.01; Table 3). There were no significant changes to adiposity from preconception to pregnancy for any diet group. In parallel to changes in adiposity, glucose-fed mice had significantly lower percentage lean mass compared to chow-fed mice at preconception, and to both chow and fructose-fed mice during pregnancy (data not shown; preconception: chow *p* < 0.01; pregnancy: chow *p* < 0.001, fructose *p* < 0.01). Only glucose and chow-fed dams had decreased percentage lean mass during pregnancy compared to at preconception (chow *p* < 0.001; glucose *p* < 0.05).

**TABLE 3:** Dam adiposity, triglyceride levels, and glucose homeostasis before and during pregnancy in Part 2 (glucose vs. fructose).

	*Timepoint*	Chow	Glucose	Fructose
Total body fat (%)	*Preconception*	11.2 ± 0.9	19.7 ± 2.3**	15.0 ± 1.6
Total body fat (%)	*Mid-gestation*	12.7 ± 0.7	23.1 ± 1.8***	16.5 ± 1.4^##^
Subcutaneous WAT mass (g)	*30 weeks of age*	0.28 ± 0.02	0.82 ± 0.09***	0.59 ± 0.07*^#^
Ovarian WAT mass (g)	*30 weeks of age*	0.31 ± 0.03	1.16 ± 0.17**	0.82 ± 0.12*
Retroperitoneal WAT mass (mg)	*30 weeks of age*	54 ± 9	246 ± 37**	190 ± 37*
Intrascapular BAT mass (mg)	*30 weeks of age*	57 ± 5	111 ± 13**	65 ± 6^##^
Plasma triglyceride (μmol/μl)	*At term*	2.5 ± 0.2	1.3 ± 0.4**	1.1 ± 0.1**
Plasma triglyceride (μmol/μl)	*30 weeks of age*	2.3 ± 0.1	1.5 ± 0.1**	1.4 ± 0.1**
Liver triglyceride (μmol/g)	*At term*	27 ± 5	32 ± 2	46 ± 3**^#^
Liver triglyceride (μmol/g)	*30 weeks of age*	25 ± 4	51 ± 2*^‡⁣‡^	46 ± 5*
Basal blood glucose (mM)	*Preconception*	8.9 ± 0.4	9.2 ± 0.3	8.8 ± 0.3
Basal blood glucose (mM)	*Mid-gestation*	9.2 ± 0.3	9.6 ± 0.4	10.1 ± 0.3^†^
Fasting plasma insulin	*Preconception*	0.3 ± 0.05	0.6 ± 0.08*	0.5 ± 0.10
Fasting plasma insulin (ng/mL)	*Mid-gestation*	0.7 ± 0.08^†⁣†^	1.3 ± 0.14**^†⁣†⁣†^	1.1 ± 0.24^†⁣†^
30 min insulin	*Preconception*	0.6 ± 0.08	1.0 ± 0.14*	0.8 ± 0.11
30 min insulin	*Mid-gestation*	1.0 ± 0.12^†^	1.8 ± 0.32*^†^	1.6 ± 0.30^†^

Dam percentage fat mass, basal fasting blood glucose and plasma insulin, and 30 min insulin in response to an oral glucose tolerance test before and during pregnancy n = 8–10 per diet; dam adipose depot weights at 30 weeks of age n = 4–6 per diet; dam triglyceride levels in plasma and liver at day E18 of pregnancy or 30 weeks of age n = 3–5 per diet per timepoint. Data presented as mean ± SEM. *p < 0.05 vs. chow; ^**^p < 0.01 vs. chow; ***p < 0.001 vs. chow; ^#^p < 0.05 vs. glucose; ^##^p < 001 vs. glucose; ^†^p < 0.05 vs. preconception for the same diet; ^†⁣†^p < 0.01 vs. preconception for the same diet; ^†⁣†⁣†^p < 0.001 vs. preconception for the same diet; ^‡⁣‡^p < 0.01 vs. at term for the same diet. WAT, white adipose tissue; BAT, brown adipose tissue.

Expressed as absolute tissue weight, glucose-fed dams at 30 weeks of age had larger subcutaneous WAT depots than both chow and fructose-fed mice, with fructose-fed mice also having more subcutaneous WAT than chow-fed mice (glucose: *p* < 0.001 vs. chow, *p* < 0.05 vs. fructose; fructose: *p* < 0.05 vs. chow; Table 3). Similarly, both glucose and fructose-fed mice had more ovarian and retroperitoneal WAT than chow-fed mice (glucose *p* < 0.01, fructose *p* < 0.05, for both adipose depots). Glucose-fed mice also had more BAT than both chow and fructose-fed mice (*p* < 0.01 for both).

Glucose and fructose-fed mice had lower plasma triglyceride levels than chow-fed mice at both day E18 of pregnancy and at 30 weeks of age (*p* < 0.01 for all four comparisons; Table 3). As in Part 1, there were no significant differences in plasma triglyceride levels from day E18 of pregnancy to at 30 weeks of age for any diet. In contrast to plasma, fructose-fed mice had higher liver triglyceride content than both chow and glucose-fed mice at day E18 of pregnancy (chow *p* < 0.01, glucose *p* < 0.05). Additionally, both glucose and fructose-fed dams had higher liver triglyceride content than chow-fed dams at 30 weeks of age (*p* < 0.05 for both). Only glucose-fed dams had significantly more liver triglyceride content at 30 weeks of age than at term (*p* < 0.01).

##### Energy expenditure, respiratory quotient, and physical activity

There were no significant differences in mean 24h energy expenditure normalized to lean mass, either between diet groups at preconception nor during pregnancy, or between time points for any diet (chow preconception vs. pregnancy *p* = 0.058; Figures 2Bi,ii). Despite no changes in 24 h energy expenditure, physical activity, as measured by wheel running, was significantly decreased across all three diet groups in response to pregnancy (*p* < 0.001 for all; Figure 2Biv). There were no differences in wheel running between diets at either timepoint. At preconception, glucose and fructose-fed dams both had lower mean 24 h RQ than chow-fed dams, with glucose-fed dams also having a lower 24 h RQ than chow-fed dams during pregnancy (preconception: glucose *p* < 0.001, fructose *p* < 0.01; pregnancy: glucose *p* < 0.01; Figures 2Bv,vi). There were no significant differences in RQ between preconception and pregnancy for any of the diets.

##### Glucose tolerance

In response to an OGTT, pregnant fructose-fed dams had increased 15 min blood glucose levels compared to chow-fed dams (*p* < 0.05; glucose vs. chow *p* = 0.055, Figures 3Bi,ii). Fructose-fed mice also had increased basal blood glucose from preconception to pregnancy (*p* < 0.05, Table 3). As in section “Part 1,” chow-fed dams tended to have reduced 15 min peak glucose levels from preconception to pregnancy (*p* = 0.055). Glucose AUC was not different among diet groups or between time points. There was no significant effect of diet, time point, or the interaction between diet and time point, on glucose AUC from preconception to during pregnancy, as determined by a two-way ANOVA.

All diet groups increased basal blood insulin and insulin AUC during an OGTT from preconception to pregnancy (basal: chow, fructose *p* < 0.01, glucose *p* < 0.001; AUC: chow, fructose *p* < 0.05, glucose *p* < 0.01; Figures 3Biii,iv and Table 3). Glucose and fructose-fed mice also had increased blood insulin levels during pregnancy at all points throughout the OGTT, with chow-fed mice also having increased 30 min insulin, compared to the same diet group at preconception (15 min: glucose, fructose *p* < 0.05; 30 min: chow, glucose, fructose *p* < 0.05; 120 min: glucose, fructose *p* < 0.05). Glucose-fed dams had significantly higher blood insulin levels at basal and at 30 min during an OGTT than chow-fed dams, both at preconception and during pregnancy (preconception: basal, 30 min p < 0.05; pregnancy: basal *p* < 0.01, 30 min *p* < 0.05, 120 min *p* = 0.051). Also during pregnancy, both glucose and fructose-fed mice had increased insulin AUC compared to chow-fed mice (*p* < 0.05 for both). As shown by a two-way ANOVA, there was a significant effect of time point, but not diet nor the interaction of diet and time point, on insulin AUC from preconception to pregnancy (*p* < 0.001).

### Pups

#### Fetal weight and organ size

##### Part 1 (sucrose vs. isomaltulose)

**FIGURE 4:**
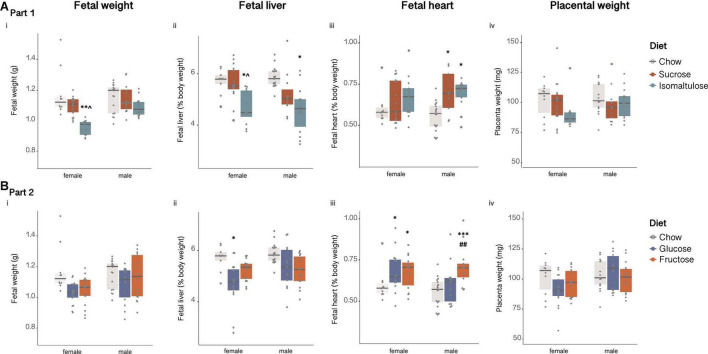
Female and male fetal **(A)** in Part 1 and **(B)** in Part 2: (i) body weight, (ii) percentage liver weight, (iii) percentage heart weight and, (iv) placental weight. *n* = 9–18 per diet per sex. *p < 0.05 vs. chow; **p < 0.01 vs. chow; ***p < 0.001 vs. chow; ^*p* < 0.05 vs. sucrose; ^##^*p* < 0.01 vs. glucose.

Female fetuses of isomaltulose-fed mothers weighed significantly less than those of both chow and sucrose-fed mothers (chow *p* < 0.01; sucrose *p* < 0.05; Figure 4Ai). There were no significant differences between male fetuses in body weight.

Female fetuses of isomaltulose-fed mothers also had smaller livers, expressed as absolute mass and as percentage of body weight, than those of chow and sucrose-fed mothers (*p* < 0.05 for both; Figure 4Aii). In male fetuses, those of isomaltulose-fed mothers also had smaller livers than male fetuses of chow-fed dams (*p* < 0.05). Male sucrose and isomaltulose-fed fetuses had larger heart mass, and as a percentage of body weight, than those of chow-fed male fetuses (*p* < 0.05 for both; Figure 4Aiii). There were no differences in female fetus heart size.

Similar to fetal weight, the placentas of female isomaltulose-fed fetuses were smaller than those of both chow and sucrose-fed female fetuses (chow *p* < 0.01; sucrose *p* < 0.05; Figure 4Aiv). There were no significant differences in male fetus placental weight.

##### Part 2 (glucose vs. fructose)

There were no significant differences in fetal body weight, or in placental weight, between diet groups for either female or male fetuses (Figures 4Bi,iv).

However, female fetuses of glucose-fed mothers had smaller livers, expressed as absolute mass and as a percentage of body weight, than those of chow-fed mothers (*p* < 0.05; Figure 4Bii). There were no differences in male fetal liver size. Male fructose-fed fetuses had larger hearts as a percentage of body weight than those of both chow and glucose-fed male fetuses (chow *p* < 0.001; glucose *p* < 0.01; Figure 4Biii). Both female glucose and fructose-fed fetuses had larger hearts than chow-fed female fetuses (*p* < 0.05 for both).

#### Part 1 (sucrose vs. isomaltulose)

##### Body weight

**FIGURE 5:**
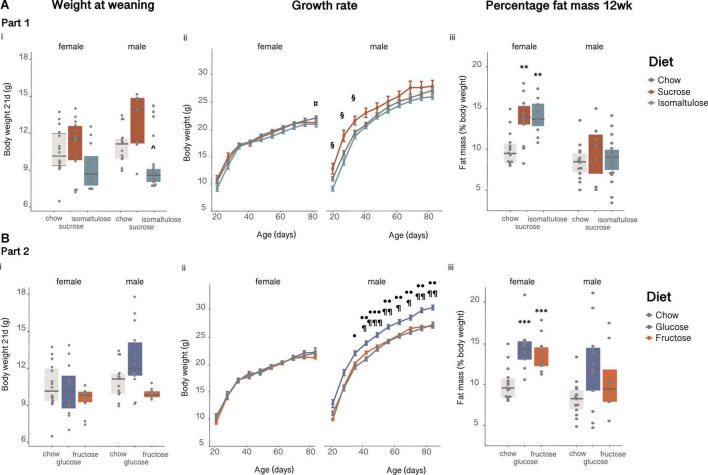
Female and male pup **(A)** in Part 1 and **(B)** in Part 2: (i) body weight at weaning, (ii) body weight over time from 3 to 12 weeks of age and, (iii) percentage body fat at 12 weeks. *n* = 7–23 per diet per sex. Line graphs depict data as mean ± SEM. **p < 0.01 vs. chow; ***p < 0.001 vs. chow; ^*p* < 0.05 vs. sucrose; ¤*p* < 0.05 isomaltulose vs. chow; § *p* < 0.05 sucrose vs. isomaltulose; ^⋅^*p* < 0.05 glucose vs. chow; ^⋅⋅^*p* < 0.01 glucose vs. chow; ^⋅⋅⋅^*p* < 0.001 glucose vs. chow; ¶ *p* < 0.05 glucose vs. fructose; ¶¶ *p* < 0.01 glucose vs. fructose; ¶¶¶ *p* < 0.001 glucose vs. fructose.

Isomaltulose-fed pups were smaller than chow-fed pups at birth (data not shown; *p* < 0.05). There were no significant differences in female pup weaning weight (Figure 5Ai). In males, isomaltulose-fed pups were smaller than sucrose-fed male pups at weaning (*p* < 0.05). Isomaltulose-fed males remained smaller than sucrose-fed males up to 5 weeks of age, but this difference disappeared by 6 weeks of age (*p* < 0.05; Figure 5Aii). There were no differences in female pup body weight up to 11 weeks of age, but at 12 weeks of age isomaltulose-fed females were significantly smaller than chow-fed females (*p* < 0.05; sucrose vs. chow *p* = 0.052). As determined by a two-way ANOVA, there was an effect of both age and diet on body weight for both male and female pups, but there was only a significant effect of the interaction of age and diet for male pups (*p* < 0.001 for all five comparisons).

##### Body fat and triglyceride analyses

By 12 weeks of age, both sucrose- and isomaltulose-fed female pups had significantly greater adiposity, and less lean mass as a percentage of body weight, than chow-fed female pups (*p* < 0.01 for both; Figure 5Aiii). There were no significant differences in either percentage lean or fat mass for male pups.

Female sucrose and isomaltulose-fed pups were shorter than chow-fed pups (sucrose *p* < 0.01; isomaltulose *p* < 0.001; Table 4). There were no significant differences in male body length. As a percentage of total body weight, both female and male sucrose-fed pups had larger subcutaneous WAT depots than chow-fed females and males, respectively (female *p* < 0.01; male *p* < 0.05). Male sucrose-fed pups also had more gonadal WAT than chow-fed male pups (*p* < 0.05). In contrast, isomaltulose-fed female pups had more gonadal WAT than chow-fed females (*p* < 0.05). There were no significant differences in BAT weight between either female or male pup diet groups.

**TABLE 4:** Pup adiposity, triglyceride levels, and glucose homeostasis at 12 weeks of age in Part 1 (sucrose vs. isomaltulose).

	*Sex*	Chow	Sucrose	Isomaltulose
Body length (cm)	*Female*	9.7 ± 0.07	9.4 ± 0.06**	9.3 ± 0.10***
Body length (cm)	*Male*	10.0 ± 0.04	9.9 ± 0.16	9.8 ± 0.05
Subcutaneous WAT (% body weight)	*Female*	0.9 ± 0.05	1.2 ± 0.04**	1.0 ± 0.06
Subcutaneous WAT (% body weight)	*Male*	0.7 ± 0.04	1.3 ± 0.30*	0.9 ± 0.04
Gonadal WAT (% body weight)	*Female*	0.8 ± 0.09	1.1 ± 0.04	1.1 ± 0.11*
Gonadal WAT (% body weight)	*Male*	1.2 ± 0.05	1.6 ± 0.21*	1.4 ± 0.07
Intrascapular BAT (% body weight)	*Female*	0.23 ± 0.01	0.29 ± 0.01	0.29 ± 0.02
Intrascapular BAT (% body weight)	*Male*	0.22 ± 0.01	0.33 ± 0.04	0.37 ± 0.07
Plasma triglyceride (μmol/μl)	*Female*	1.1 ± 0.2	1.1 ± 0.3	0.9 ± 0.2
Plasma triglyceride (μmol/μl)	*Male*	1.8 ± 0.3	1.2 ± 0.5	1.0 ± 0.2
Liver triglyceride (μmol/g)	*Female*	20 ± 2	34 ± 5*	21 ± 2^
Liver triglyceride (μmol/g)	*Male*	16 ± 2	29 ± 12	14 ± 2
Leptin (ng/ml)	*Female*	1.6 ± 0.8	2.3 ± 0.4*	1.9 ± 0.3
Leptin (ng/ml)	*Male*	2.4 ± 1.2	10.8 ± 4.8	2.5 ± 1.0
Basal blood glucose (mM)	*Female*	8.6 ± 0.3	8.4 ± 0.5	8.3 ± 0.3
Basal blood glucose (mM)	*Male*	10.1 ± 0.3	10.2 ± 0.3	9.2 ± 0.3
Fasting plasma insulin (ng/mL)	*Female*	0.4 ± 0.05	0.7 ± 0.10**	0.5 ± 0.04
Fasting plasma insulin	*Male*	0.7 ± 0.06	0.8 ± 0.17	0.8 ± 0.09
30 min insulin	*Female*	0.4 ± 0.07	0.9 ± 0.09	1.0 ± 0.20
30 min insulin	*Male*	0.8 ± 0.09	1.1 ± 0.11	1.7 ± 0.4

Female and male pup body length and adipose weights at 12 weeks of age n = 6–23 per diet per sex; pup triglyceride levels in plasma and liver n = 7–23 per diet per sex; pup fasting plasma leptin n = 6–8 per diet per sex; pup basal fasting glucose and plasma insulin, and 30 min insulin in response to an oral glucose tolerance test n = 7–21 per diet per sex. Data presented as mean ± SEM. *p < 0.05 vs. chow; **p < 0.01 vs. chow; ***p < 0.001 vs. chow; ^p < 0.05 vs. sucrose. WAT, white adipose tissue; BAT, brown adipose tissue.

There were no significant differences detected in plasma triglyceride levels in either female or male pups (Table 4). Sucrose-fed female pups had higher liver triglyceride content than both chow and isomaltulose-fed pups (*p* < 0.05 for both). Sucrose-fed female pups also had increased fasting circulating leptin levels compared to chow-fed female pups (*p* < 0.05). There were no significant differences in either male pup liver triglyceride levels or plasma leptin levels (liver triglyceride: sucrose vs. isomaltulose *p* = 0.053).

##### Energy expenditure, respiratory quotient, and physical activity

**FIGURE 6:**
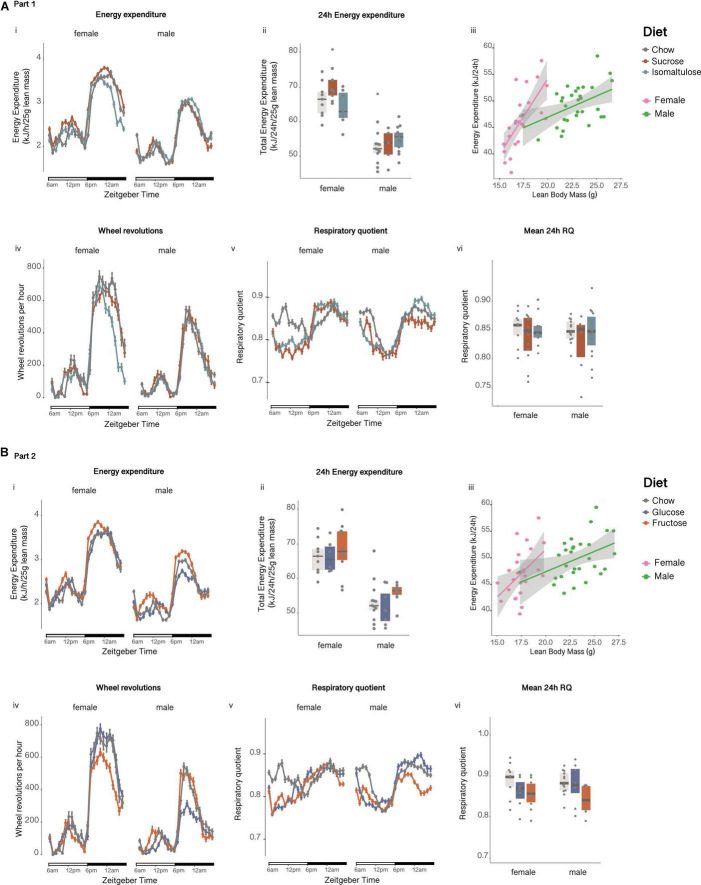
Metabolic phenotyping of female and male pups at 12 weeks of age **(A)** in Part 1 and **(B)** in Part 2: Pup (i) energy expenditure over time, (ii) total energy expenditure, (iii) correlation of energy expenditure and lean mass, (iv) wheel revolutions, (v) respiratory quotient over time and, (vi) respiratory quotient. *n* = 10–26 per diet per sex. Line graphs depict data as mean ± SEM.

There were no significant differences in either female or male mean 24 h energy expenditure normalized to lean mass at 12 weeks of age (Figures 6Ai,ii). There were also no significant differences in female or male physical activity or wheel running (Figure 6Aiv). Furthermore, there were no significant differences in mean 24 h RQ between diets for either sex (Figures 6Av,vi).

##### Glucose tolerance

**FIGURE 7:**
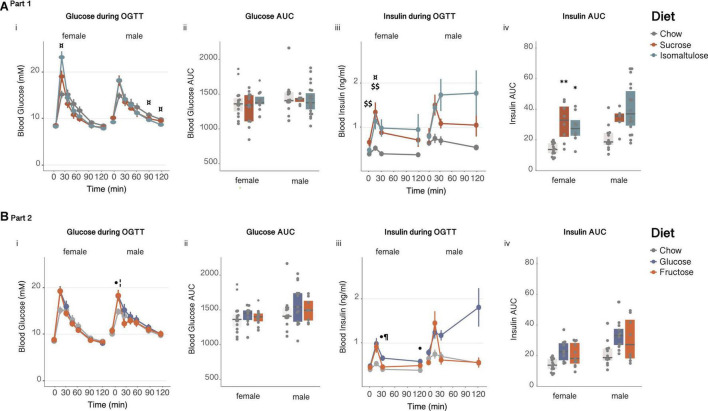
Glucose homeostasis in pups **(A)** in Part 1 and **(B)** in Part 2: Female and male pup response to an oral glucose tolerance test at 12 weeks of age (i) glucose over time, (ii) glucose AUC, (iii) insulin over time and, (iv) insulin AUC. *n* = 7–21 per diet per sex. Line graphs depict data as mean ± SEM. *p < 0.05 vs. chow; **p < 0.01 vs. chow; ^$$^*p* < 0.01 sucrose vs. chow; ¤*p* < 0.05 isomaltulose vs. chow; ^⋅^*p* < 0.05 glucose vs. chow; ¦*p* < 0.05 fructose vs. chow; ¶ *p* < 0.05 glucose vs. fructose.

There were no significant differences in basal blood glucose levels or glucose AUC during an OGTT at 12 weeks of age for either female or male pups (Figures 7Ai,ii and Table 4). Female isomaltulose-fed pups had increased 15 min blood glucose levels compared to chow-fed females (*p* < 0.05; isomaltulose vs. sucrose *p* = 0.056). In contrast, male isomaltulose-fed pups had lower 90 and 120 min glucose levels compared to chow-fed male pups (*p* < 0.05 for both).

Twelve-week-old female sucrose-fed pups had higher basal insulin levels than chow-fed females (*p* < 0.01; Figure 7Aiii and Table 4). Both sucrose and isomaltulose-fed female pups had a higher insulin AUC in response to an OGTT than chow-fed female pups (sucrose *p* < 0.01; isomaltulose *p* < 0.05; Figure 7Aiv). Sucrose and isomaltulose-fed female pups also had higher 15 min insulin levels than chow-fed female pups (sucrose *p* < 0.01; isomaltulose *p* < 0.05). There were no significant differences detected in insulin response to an OGTT between diet groups in male pups at 12 weeks of age.

#### Part 2 (glucose vs. fructose)

##### Body weight

There were no significant differences in pup birth weight, or in weaning weight between the diet groups for either sex (birth data not shown; Figure 5Bi). There were also no significant differences in female pup body weight at any time point (Figure 5Bii). Despite no differences in birth or weaning weight, glucose-fed males were significantly heavier than chow-fed males at 5 weeks of age, and remained heavier than both chow and fructose-fed males from 6 weeks of age (*p* < 0.05). There was a significant effect of both age and diet, as well as a significant effect of the interaction of age and diet, on body weight for female pups (diet *p* < 0.01; age *p* < 0.001; interaction *p* < 0.05). For male pups, there was a significant effect of both age and diet on body weight but no significant interaction (*p* < 0.001 for both).

##### Body fat and triglyceride analyses

As in Part 1, at 12 weeks of age, both sugar-diet female pup groups had significantly higher body fat percentage and significantly lower percentage lean mass than chow-fed female pups (fat: *p* < 0.001 for both; lean: *p* < 0.01 for both; Figure 5Biii). Also similar to Part 1, there were no significant differences in either percentage lean or fat mass for male pups.

Male glucose-fed pups were longer than chow and fructose-fed male pups (chow *p* < 0.05; fructose *p* < 0.01; Table 5). There were no significant differences in female body length. Both female glucose and fructose-fed pups had larger subcutaneous WAT depots as a percentage of body weight compared to chow-fed females (*p* < 0.05 for both). Male glucose-fed pups had more subcutaneous WAT than chow-fed males (*p* < 0.05). Despite no differences in size, female glucose-fed pups had larger gonadal WAT depots than female pups of both other diets, with fructose-fed females also having more gonadal WAT than chow-fed females (chow *p* < 0.001, fructose *p* < 0.05 vs. glucose; fructose *p* < 0.05 vs. chow). There were no significant differences in male gonadal WAT. Both female and male glucose-fed pups had larger BAT depots than chow-fed pups of the same sex, with fructose-fed females also having more BAT than chow-fed females (*p* < 0.05 for all three comparisons).

**TABLE 5:** Pup adiposity, triglyceride levels, and glucose homeostasis at 12 weeks of age in Part 2 (glucose vs. fructose).

	*Sex*	Chow	Glucose	Fructose
Body length (cm)	*Female*	9.7 ± 0.07	9.4 ± 0.10	9.5 ± 0.09
Body length (cm)	*Male*	10.0 ± 0.04	10.1 ± 0.08*	9.9 ± 0.06^##^
Subcutaneous WAT (% body weight)	*Female*	0.9 ± 0.05	1.2 ± 0.07*	1.1 ± 0.07*
Subcutaneous WAT (% body weight)	*Male*	0.7 ± 0.04	1.1 ± 0.10*	0.9 ± 0.09
Gonadal WAT (% body weight)	*Female*	0.8 ± 0.09	1.4 ± 0.11***	1.1 ± 0.07*^#^
Gonadal WAT (% body weight)	*Male*	1.2 ± 0.05	1.9 ± 0.21	1.7 ± 0.15
Intrascapular BAT (% body weight)	*Female*	0.23 ± 0.01	0.34 ± 0.07*	0.28 ± 0.01*
Intrascapular BAT (% body weight)	*Male*	0.22 ± 0.01	0.29 ± 0.02*	0.27 ± 0.02
Plasma triglyceride (μmol/μl)	*Female*	1.1 ± 0.2	0.8 ± 0.1	1.0 ± 0.3
Plasma triglyceride (μmol/μl)	*Male*	1.8 ± 0.3	1.1 ± 0.2	0.7 ± 0.1
Liver triglyceride (μmol/g)	*Female*	20 ± 2	34 ± 2**	25 ± 3^#^
Liver triglyceride (μmol/g)	*Male*	16 ± 2	23 ± 3	14 ± 4
Leptin (ng/ml)	*Female*	1.6 ± 0.8	2.1 ± 0.4*	3.4 ± 1.4*
Leptin (ng/ml)	*Male*	2.4 ± 1.2	6.6 ± 3.6	3.6 ± 1.0
Basal blood glucose (mM)	*Female*	8.6 ± 0.3	8.8 ± 0.3	8.9 ± 0.5
Basal blood glucose (mM)	*Male*	10.1 ± 0.3	10.8 ± 0.5	10.8 ± 0.7
Fasting plasma insulin	*Female*	0.4 ± 0.05	0.5 ± 0.05	0.4 ± 0.04
Fasting plasma insulin	*Male*	0.7 ± 0.06	0.9 ± 0.17	0.5 ± 0.06
30 min insulin	*Female*	0.4 ± 0.07	0.7 ± 0.06*	0.5 ± 0.05^#^
30 min insulin	*Male*	0.8 ± 0.09	1.3 ± 0.14	0.6 ± 0.11

Female and male pup body length and adipose weights at 12 weeks of age n = 6–23 per diet per sex; pup triglyceride levels in plasma and liver n = 7–23 per diet per sex; pup fasting plasma leptin n = 6–8 per diet per sex; pup basal fasting glucose and plasma insulin, and 30 min insulin in response to an oral glucose tolerance test n = 7–21 per diet per sex. Data presented as mean ± SEM. *p < 0.05 vs. chow; **p < 0.01 vs. chow; ***p < 0.001 vs. chow; ^#^p < 0.05 vs. glucose; ^##^p < 0.01 vs. glucose. WAT, white adipose tissue; BAT, brown adipose tissue.

Similar to Part 1, there were no significant differences detected in plasma triglyceride levels in either female or male pups (Table 5). Glucose-fed female pups had higher liver triglyceride content than both chow and fructose-fed pups (chow *p* < 0.01; fructose *p* < 0.05). Both female sugar-diet groups had increased fasting circulating leptin levels compared to chow-fed female pups (*p* < 0.05 for both). There were no significant differences in either male pup liver triglyceride or plasma leptin levels.

##### Energy expenditure, respiratory quotient, and physical activity

12-week-old male glucose-fed pups tended to have lower lean mass normalized 24 h energy expenditure than fructose-fed male pups (*p* = 0.056; Figures 6Bi,ii). There were no significant differences in female 24 h energy expenditure. Corroborating the decrease in energy expenditure, male glucose-fed pups were less physically active than both chow and fructose-fed males, having a lower number of average wheel revolutions per 24 h (*p* < 0.05 for both; Figure 6Biv). There were no significant differences in female physical activity or wheel revolutions. There were also no significant differences in mean 24 h RQ between diets for either sex (Figures 6Bv,vi).

##### Glucose tolerance

Similar to Part 1, there were no significant differences in basal blood glucose levels or glucose AUC during an OGTT at 12 weeks of age for either female or male pups (Figures 7Bi,ii and Table 5). Both male glucose and fructose-fed pups had higher 15 min glucose levels compared to chow-fed males (*p* < 0.05 for both). There were no significant differences in glucose levels between female diet groups at any time point during an OGTT.

There were also no significant differences in basal blood insulin levels or insulin AUC during an OGTT at 12 weeks of age for either female or male pups (Figures 7Biii,iv and Table 5). Female glucose-fed pups had a higher 30 and 120 min insulin response than chow-fed female pups, as well as a higher 30 min blood insulin level compared to fructose-fed females (*p* < 0.05 for all three comparisons). As in Part 1, there were no significant differences detected in insulin response to an OGTT between diet groups in male pups at 12 weeks of age.

## Discussion

This is the first study in mice designed to determine the effects of different saccharides and GI on maternal metabolic status, fetal development, and offspring adiposity and glucose tolerance independently of dietary energy density, macronutrient composition, and other markers of carbohydrate quality.

Female mice fed a glucose-based diet were heavier and fatter than chow and isomaltulose-fed mice before and during pregnancy. Weight at conception correlated with gestational weight gain across all groups, consistent with previous studies in humans (29, 30). However, when examined per diet group, this correlation was only significant in glucose-fed dams. Energy expenditure, physical activity, and energy intake did not vary significantly between diets, especially in the offspring. However, food intake was markedly underestimated and represented only ∼60% of total energy expended. More precise measurement of energy intake is required to resolve any effect of dietary saccharide composition on appetite which might account for differences in body weight.

The low GI isomaltulose-based diet improved oral glucose tolerance when compared with sucrose, in agreement with reported correlations of both increased body fat mass and high GI diets with glucose tolerance and insulin resistance (31–33). There were no observed differences in oral glucose tolerance between glucose and fructose-fed female mice pre-pregnancy despite the difference in body adiposity. All dams had increased insulin response to an OGTT during pregnancy, reflecting that pregnancy is an insulin resistant state, purportedly to increase glucose and nutrient transmission to the developing fetus (34). When comparing isomaltulose and sucrose-based diets, which differ only in the rate of disaccharide digestion, isomaltulose-fed dams had a lower basal blood glucose and glucose AUC preconception. This result suggests that increased postprandial glycemia, that is, GI or rate of carbohydrate digestion and absorption, in sucrose-fed dams resulted in poorer glucose tolerance. However, there were no differences in basal plasma insulin at sacrifice or at 30 min timepoint during an OGTT between isomaltulose and sucrose dietary groups.

The effect of dietary carbohydrate type on fetal and offspring measurements varied by sex. While both glucose and isomaltulose constrained female fetal growth relative to chow controls, only isomaltulose placentas were significantly smaller, suggesting the mechanisms through which fetal growth is reduced may be different. Female fetuses from isomaltulose-fed mothers were also lighter than those of sucrose-fed mothers suggesting lower postprandial glycemia, or differences in incretin responses, such as GIP and GLP1, may be important factors in determining fetal growth in mice. Birth weight is a measure of nutritional exposure *in utero* and both under- and overnutrition are linked to changes in birth weight (35). Unhealthy maternal diets characterized by high intake of refined carbohydrates, processed meat, and foods high in saturated fat are associated with lower birth weight in humans (36). Maternal obesity has also been shown to reduce fetal weight through altered placental structure (37). The primary fuel for fetal growth is glucose, prompting us to consider whether both a low and high rate of dietary glucose availability are correlated with reduced fetal size. Maternal mechanisms to limit fetal size are important to ensure survival of the mother. We speculate that in mice fed a high-isomaltulose diet, the fetal demand for glucose is unmet through dietary supply and dietary protein undergoes gluconeogenesis to maintain maternal blood glucose levels, which should be confirmed in future studies.

As a percentage of fetal weight, livers from both male and female fetuses exposed to a maternal high-isomaltulose diet, as well as livers from female fetuses exposed to a maternal high-glucose diet, were smaller than those from fetuses of chow-fed dams. Reduced liver mass may indicate constrained development of the liver as a consequence of early undernutrition. The concept of fetal brain-sparing proposes that when energy to the fetus is limited, energy is diverted toward growth of essential organs, such as the brain, at the expense of other (metabolic) tissues (38). In support of this mechanism at play in our model, fetal brain mass did not differ among dietary groups (data not shown). A smaller liver may indicate a reduced metabolic capacity and increased susceptibility to development of metabolic diseases under conditions of high metabolic load, or overnutrition postnatally (39).

Interestingly, male fetal heart mass was heavier in those of dams fed diets containing fructose (high-sucrose, -isomaltulose or -fructose diets) compared with chow or high-glucose diet groups. This is interesting because increased heart mass is a marker of stiff, impaired relaxation heart failure and represents half of all heart failures for which there are no therapies (40). An obvious question arising from these observations is whether dietary fructose contributes to the developmental origin of this type of heart disease, and whether it might explain the greater prevalence in males.

Fetal sex is an important modifier of the effect of maternal nutrition on fetal growth. Sex differences in the placenta’s response to nutrition imply different strategies of males and females for reproductive success. Female pup fetal growth rate is moderated to optimize survival in the face of an adverse postnatal environment and often display greater placental adaptation. Boys have a riskier strategy, a faster rate of fetal growth and less investment in placental growth and reserves (41). The differential sex responses during development may provide important clues in our understanding of how sex modifies risk of non-communicable diseases later in life.

Maternal obesity is another important confounding factor to consider. A maternal obese environment can stimulate the fetus to produce high levels of insulin, leptin and low levels of adiponectin, adding another layer of complexity to the intrauterine milieu, and likely confounds the maternal dietary effects on offspring in this study (42). Future studies directly comparing saccharides may want to pair-feed dams to minimize the contribution of the maternal obese state.

In 12-week-old pups fed the same diets as their mothers, high-sugar fed females were all fatter than chow-fed female offspring despite being of the same body weight. This raises the idea that the processed nature of the highly refined sugar diets compared to the minimally processed chow has a greater effect on body composition than the overall quality and rate of digestion and absorption in females.

In male offspring, only high-glucose fed pups were significantly heavier and fatter than chow fed pups, similar to the trend we saw in their mothers. This perhaps concurs with previous evidence that males are more likely to exhibit “risky” behavior, achieved through a less-selective placenta, as they do not need to be as programmed to ensure the survivability of the next generation (41, 43).

We found no differences in either sex for fasting glucose or oral glucose tolerance at 12 weeks. However, high-sugar diets tended to exacerbate the insulin response to glucose, suggesting the development of insulin resistance. Twelve weeks of age is a relatively young age to consider markers of metabolic health that interact with the aging process, as such, future studies should aim to determine impacts at a later stage of life in pups.

While all dams were initially mated at the same age, only a quarter became pregnant at the first instance. The remaining dams were mated multiple times, with rests in between, until pregnancy was successfully achieved. All dams remained on the test diets throughout and until euthanasia, but this resulted in a degree of variation in the length of time on the diet before pregnancy, with the minimum of 6 weeks and maximum of 20 weeks. However, as this variation existed across all diets, any effect caused should be equalized between groups. Nonetheless, for future studies this variation should be limited if possible.

The chow diet used here is a common standard reference diet. There are many ways in which unprocessed chow varies from the refined sugar diets, including wholegrain and fiber content, and is therefore not a suitable control for investigation of effects of isolated nutrients. As can be inferred by the pup caecum weights, which were all significantly smaller in sugar-diet offspring, regardless of sex, chow introduces extra confounders brought about by the microbiota, not seen in the ultra-processed high-sugar diets. In the future, the AIN-93G chow diet should be used as the reference diet as it is still a healthy control, but more similar in construction to the sugar-based diets used here (44).

Additionally, to isolate the effects of the maternal diet from the postnatal diet, in future studies pups could be cross-fostered onto the control diet. Alternatively, to limit the risks and practical difficulties inherent in cross-fostering, the mothers’ diets could be swapped at birth. Most studies that aim to separate the maternal and postnatal diet effects do so be switching the pups’ diet post weaning, but this does not address the effects of the maternal diet in the first few weeks after birth. As such, cross-fostering or alteration of the mothers’ diets may be a clearer alternative for future studies.

While the macronutrient compositions of these diets are within the typical range for humans, the proportion of carbohydrate provided as monosaccharides and disaccharides does not translate to human dietary examples. The diet construction was instead designed to directly compare effects of saccharides and the effect of dietary GI on developmental metabolic outcomes. Additionally, this composition was deemed necessary in order to sufficiently control for both macronutrient and fiber content. In a systematic review and meta-analysis of high GI rodent diets we previously conducted, only one study controlled for both macronutrient and fiber content (45). This singular study also used high-sugar diets, based on glucose and fructose, similarly to those we implemented in Part 2 (46). However, recent studies suggest that at > 60% energy contribution to the diet, fructose saturates intestinal metabolism leading to spill over of fructose to liver and colonic microbiota, indicating the phenotype of these mice may be influenced by microbiota-associated effects (47). Although fructose has a low GI, it clearly has GI-independent effects on rodents that are detrimental when fed in excess.

## Conclusion

The effect of carbohydrate quality on offspring development and metabolic programming has little been studied. High-glucose diets led to greater adiposity and body weight in dams and their male offspring compared with chow-fed controls. Female fetuses of dams fed high-glucose or -isomaltulose were smaller than those of chow-fed dams. The mechanisms for these effects are perhaps distinct from one another but further research is needed. At 12 weeks of age, female pups of all high-sugar diets were fatter than those on chow despite being of the same body weight. Sex-dependent phenotypic patterns emerging in response to carbohydrate quality will provide important advances in our understanding of how sex modifies risk of diet-related non-communicable diseases in humans. Future studies should also be conducted to create a broader cardiometabolic profile of offspring for a longer duration, following on from those results seen here. In conclusion, while low GI diets are typically beneficial for metabolic health, as shown in Part 1, perhaps the more important consideration is what makes the diet low GI; as the lowest GI diet, fructose, is still disadvantageous, as seen in Part 2.

## Data availability statement

The raw data supporting the conclusions of this article will be made available by the authors, without undue reservation.

## Ethics statement

The animal study was reviewed and approved by the University of Sydney Animal Ethics Committee, University of Sydney, Camperdown, Australia.

## Author contributions

KB-A conceived the study. GJC and KB-A designed the study. GJC, SLF, AB, and KB-A conducted the study. GJC, SLF, AS, and KB-A analyzed the results. GJC, SLF, and KB-A wrote the manuscript. All authors approved the manuscript.

## Conflict of interest

The authors declare that the research was conducted in the absence of any commercial or financial relationships that could be construed as a potential conflict of interest.

## Publisher’s note

All claims expressed in this article are solely those of the authors and do not necessarily represent those of their affiliated organizations, or those of the publisher, the editors and the reviewers. Any product that may be evaluated in this article, or claim that may be made by its manufacturer, is not guaranteed or endorsed by the publisher.

## Abbreviations

AUC: area under the curve
BAT: brown adipose tissue
GI: glycemic index
OGTT: oral glucose tolerance test
RQ: respiratory quotient
WAT: white adipose tissue.
