# Erratum: Sex-specific effects of maternal dietary carbohydrate quality on fetal development and offspring metabolic phenotype in mice

**DOI:** 10.3389/fnut.2022.1094120

**Published:** 2022-11-29

**Authors:** Frontiers Production Office

**Affiliations:** Frontiers Media SA, Lausanne, Switzerland

**Keywords:** maternal diet, glycemic index, carbohydrate quality, metabolism, mice

Due to a production error, there was a mistake in Figure 1 as published. The line thickness in Figure 1A was too thick. The corrected Figure 1 appears below.

**Figure 1:**
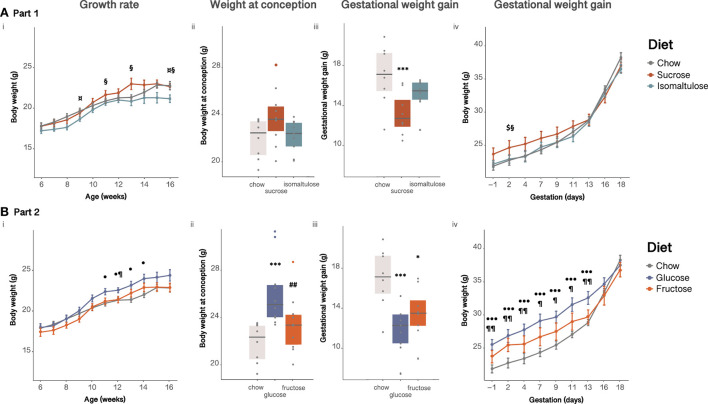
Dam body weight **(A)** in Part 1 and **(B)** in Part 2: (i) pre-pregnancy, (ii) at conception, (iii) gained during pregnancy, (iv) during pregnancy. *n* = 8–10 per diet. Line graphs depict data as mean ± SEM. ^*^p < 0.05 vs. chow; ^***^p < 0.001 vs. chow; ^##^*p* < 0.01 vs. glucose; ^$^*p* < 0.05 sucrose vs. chow; ¤*p* < 0.05 isomaltulose vs. chow; § *p* < 0.05 sucrose vs. isomaltulose; ^·^*p* < 0.05 glucose vs. chow; ^···^*p* < 0.001 glucose vs. chow; ¶ *p* < 0.05 glucose vs. fructose; ¶¶ *p* < 0.01 glucose vs. fructose.

The publisher apologizes for this mistake. The original article has been updated.

