# Supplementary material for: Sex-specific effects of maternal dietary carbohydrate quality on fetal development and offspring metabolic phenotype in mice
Source: Front Nutr. 2022 Jul 22;9:917880. doi: 10.3389/fnut.2022.917880 (PMC9356227; doi:10.3389/fnut.2022.917880)

Supplementary Material

In-house dietary ingredients’ information, supplemental to **Table 1**

| Ingredient | Supplier | Catalog Number |
| --- | --- | --- |
| Maltodextrin | Myopure | MALT2 |
| Glucose | Thermo Fisher | AJA783 |
| Sucrose | Coles | 5422530P |
| Isomaltulose | Myopure | PALATINOSE |
| Fructose | Lotus | 9317127060611 |
| Calcium Caseinate | MPD Dairy Products | T200 |
| Pressed Safflower Oil | Pressed Purity | OISA250 |
| Organic Wheat Bran | Lotus | 9317127638308 |
| AIN-93 G-MX Mineral Mix | MP Biomedicals | 0296040002 |
| Gelatine Powder | Wards McKenzie’s | 9083120P |
| AIN-93-VX Vitamin Mix | MP Biomedicals | 0296040201 |
| Choline Bitartrate | Sigma | C1629 |
| DL-Methionine | MP Biomedicals | 190955 |

Specialty Feeds Chow reference diet information from the manufacturer’s data sheet.


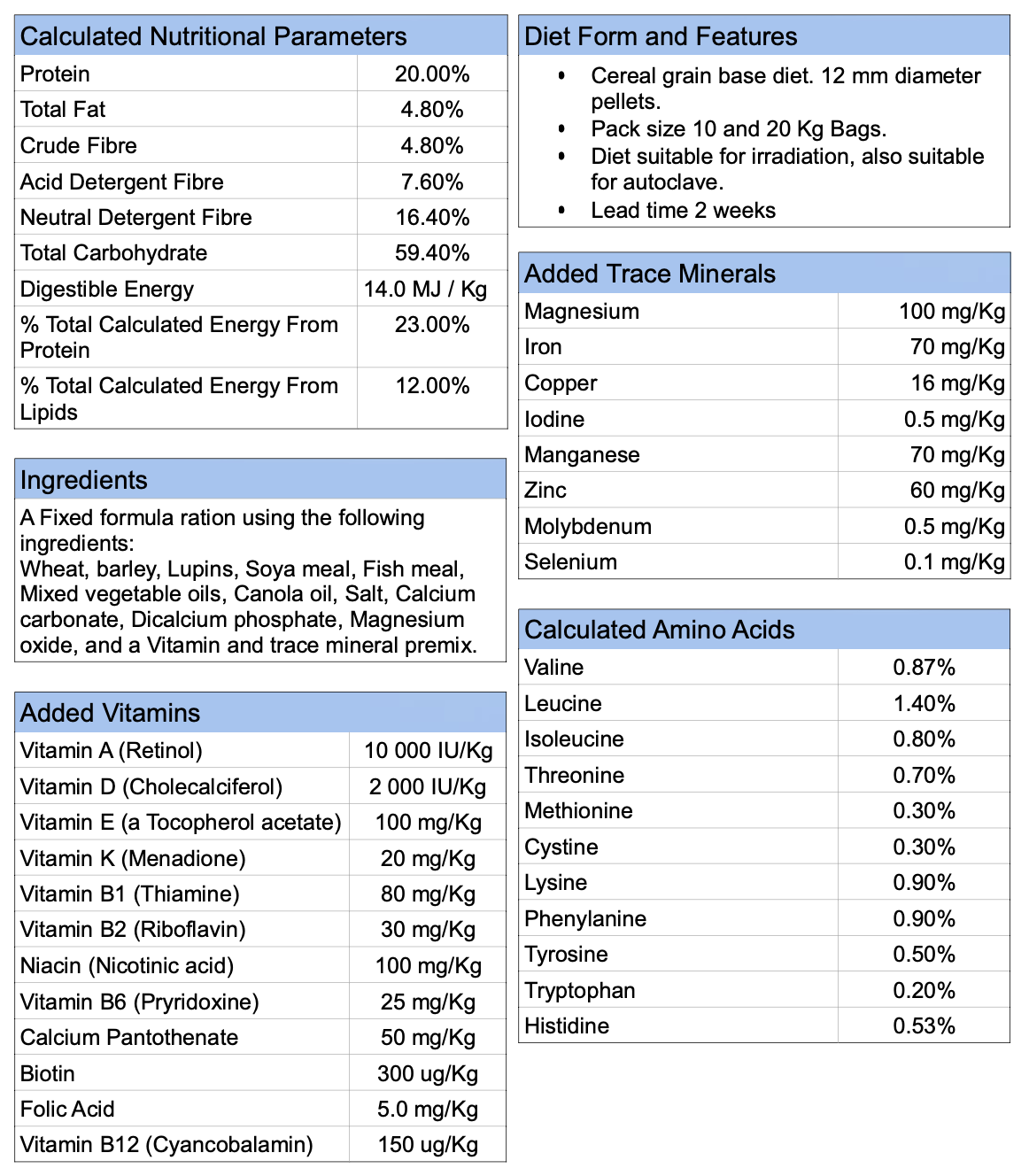


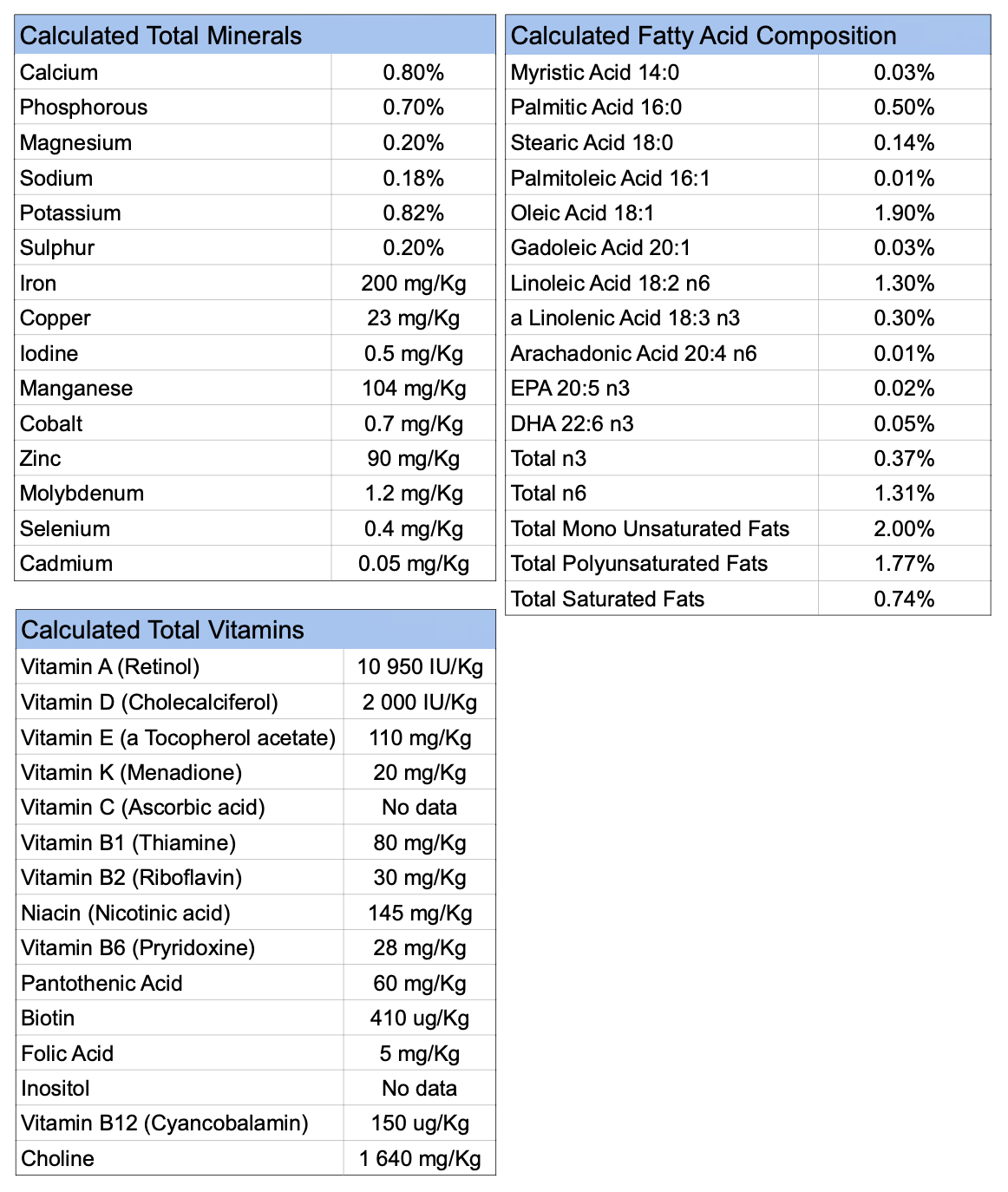

Supplement: Supplementary file 1 [file Data_Sheet_1.DOCX]
